# New insight into circRNAs: characterization, strategies, and biomedical applications

**DOI:** 10.1186/s40164-023-00451-w

**Published:** 2023-10-12

**Authors:** Xin-Yi Feng, Shun-Xin Zhu, Ke-Jia Pu, Heng-Jing Huang, Yue-Qin Chen, Wen-Tao Wang

**Affiliations:** MOE Key Laboratory of Gene Function and Regulation, Guangdong Province Key Laboratory of Pharmaceutical Functional Genes, State Key Laboratory of Biocontrol, School of Life Sciences, Sun Yat-Sen University, Guangzhou, 510275 People’s Republic of China

**Keywords:** circRNA, Database, Strategy, Therapeutic approaches, Biomedical applications

## Abstract

Circular RNAs (circRNAs) are a class of covalently closed, endogenous ncRNAs. Most circRNAs are derived from exonic or intronic sequences by precursor RNA back-splicing. Advanced high-throughput RNA sequencing and experimental technologies have enabled the extensive identification and characterization of circRNAs, such as novel types of biogenesis, tissue-specific and cell-specific expression patterns, epigenetic regulation, translation potential, localization and metabolism. Increasing evidence has revealed that circRNAs participate in diverse cellular processes, and their dysregulation is involved in the pathogenesis of various diseases, particularly cancer. In this review, we systematically discuss the characterization of circRNAs, databases, challenges for circRNA discovery, new insight into strategies used in circRNA studies and biomedical applications. Although recent studies have advanced the understanding of circRNAs, advanced knowledge and approaches for circRNA annotation, functional characterization and biomedical applications are continuously needed to provide new insights into circRNAs. The emergence of circRNA-based protein translation strategy will be a promising direction in the field of biomedicine.

## Background

CircRNA was originally regarded as incorrect RNA cleavage products in viroids [1]. With the development of high-throughput sequencing technologies, an increasing number of circRNAs have been discovered and have received much attention [2, 3]. Unlike other well-known classes of linear RNAs, such as messenger RNA (mRNA), long noncoding RNA (lncRNA), small nucleolar RNA (snoRNA), microRNA (miRNA), etc., circular RNAs are covalently closed single-stranded RNAs (ssRNAs) that have recently become a widespread class of RNA species [3–8]. Although there is still a challenge to identify and annotate novel emerging circRNAs, advances in bioinformatics algorithms, detection methods, and molecular biological techniques have provided new opportunities to accelerate the understanding of circRNAs.

In recent years, several key characterizations of circRNAs have been identified [5, 9]. Although a few circRNAs were first identified during intron self-splicing from ribosomal RNAs, mitochondrial RNAs, and tRNAs, most annotated circRNAs are generated from pre-mRNA back-splicing [4, 5, 10, 11]. In this uncommon pre-mRNA splicing, a downstream 5′ splice site is joined to an upstream 3′ splice site to form circular RNAs with a 3′,5′-phosphodiester bond at the back-splicing junction site (BSJ) [4]. Many regulators have been revealed to improve circRNA biogenesis, including intronic complementary sequences (ICSs) in flanking introns of circle-forming exons, Alu elements and RNA-binding proteins (RBPs) [4, 10, 12–14]. Due to the lower efficiency of back-splicing than that of canonical splicing, the examined cells and tissues usually showed a generally low abundance of circRNAs. Once produced, the unique covalently closed conformation of circRNAs endows them with considerable stability and more resistance to RNase R than linear RNAs [15], which enables them to regulate cellular processes with a small number of molecules. Interestingly, there are some insights into circRNA clearance, including circRNA degradation by RNase H1 in circRNA:DNA hybrids [16, 17], endonuclease RNase L during innate immune response activation [18], and the RNaseP/MRP complex in m6A modification [19]. circRNA levels can also be reduced in cancer cells with a rapid proliferation rate [9, 20].

In the past few years, circRNAs have been regarded as competing endogenous RNAs that sponge miRNAs that silence their target genes [4, 10, 21, 22]. Recent studies have revealed that circRNAs perform cellular functions via several novel regulatory mechanisms, including circRNA-RBP [23], circRNA:DNA hybrids [16, 17], m6A modification [19, 24–26], guiding A-to-I editing [27, 28], and translation potential [29–32]. These features illustrated that circRNAs may comprehensively play important roles in pathological and physiological processes. Increasing evidence indicates that circRNAs are closely associated with proliferation, metastasis, DNA damage, drug resistance and other life activities of cancer cells [20, 33–35].

Given that circRNAs have structural stability advantages and that the negative effect of intron-derived circRNAs on triggering the immune response is smaller than that of other RNAs, the development of RNA drugs based on circRNAs has important application prospects [5, 9, 36]. circRNAs can be relatively stable in biological fluids and may serve as good biomarkers for early diagnosis and prognosis [36, 37]. Several tissue-specific circRNAs have been suggested to be used as targets for cancer treatment, even in therapy resistance and targeted drug development [38–43]. Of note, RNA circle-based translation technologies have emerged as a promising strategy in biomedicine [9, 30, 44, 45]. For example, the circRNA-RBD-Delta vaccine was designed to resist the COVID-19 pandemic [44].

In this review, we collected the recent progress in the biogenesis, degradation and biology of circRNAs and describe novel technologies for the identification, accurate quantification, and functional characterization of circRNAs. Based upon our findings, we also discuss the current challenges of circRNA analysis and new insight into strategies to determine circRNA functions and the biomedical implications of circRNA.

## Characterization of circRNAs

### Biogenesis of circRNAs

**Fig. 1:**
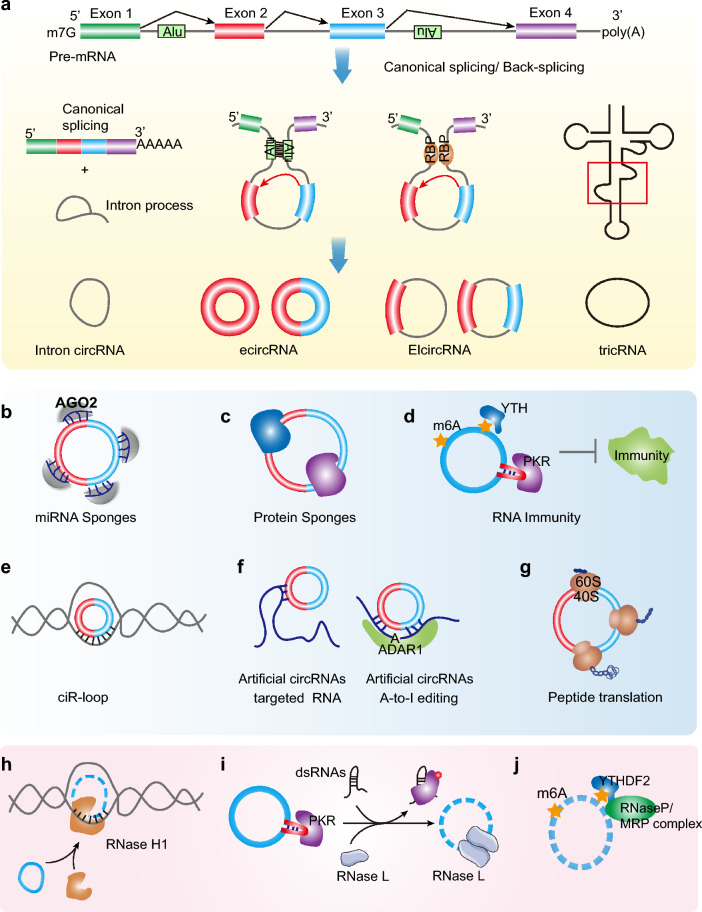
Characterization of circRNAs. **a** Biogenesis of circRNAs. circRNAs are produced from back-splicing or intron self-splicing of precursor RNAs. Exons, introns, 5′untranslated regions (UTR), 3′UTR or antisense sequences, can generate circRNAs, which include ciRNAs, EIciRNAs, ecircRNAs, and others. CircRNAs regulate biological process via various regulation mechanism, including circRNA-miRNA sponge (**b**), circRNA-protein interaction (**c**), circRNA immunity (**d**), circR-loop (**e**), guiding A-to-I editing (**f**), and translation (**g**). **h**, **i** Degradation mechanism of circRNA. **h** RNase H1 degrades a subgroup of ciRNAs in R-loops. **i** circRNAs are degraded by RNase L in PKR activated innate immunity. **j** Recruitment of endonucleases by m6A marks to degrade circRNAs

In general, circular RNA is usually derived from back-splicing of pre-mRNA to form a closed RNA transcript [3, 5, 10, 11]. Additionally, circular RNA can intermediately originate from small nuclear RNAs (snRNAs), mitochondrial RNAs, ribosomal RNAs (rRNAs), and transfer RNAs (tRNAs) during intron self-splicing [5, 42, 46–48]. Advancing RNA sequencing (RNA-seq) technologies and computational pipelines for circular RNA annotation, recent studies have found that circRNAs can be derived from exons, introns, 5' untranslated regions (UTRs), 3' UTRs or antisense sequences and can be classified into four main categories, intronic circRNAs (ciRNAs), exon‒intron circRNAs (EIciRNAs), exonic circRNAs (ecircRNAs), and others, detected in a variety of organisms, including viruses, archaea, plants, parasites, and most mammals [4, 5, 10, 11, 49, 50] (Fig. 1a). Evidence has shown that back-splicing of pre-mRNA is the predominant process for circRNA generation [3, 50]. In this back-splicing process of pre-mRNA, a splice donor that is downstream of the 5’ splice site is joined to a splice acceptor that is upstream of the 3’ splice site, producing a circular format with a 3’-5’ phosphodiester bond at the back-splicing junction site (BSJ) [3]. In addition, RBPs, special sequences of introns, etc., may assist in the production of circRNA [3, 12, 15]. Circularized RBPs can shorten the distance between the upstream and downstream of the circular exon by connecting related intron sequences, promote splicing, and induce the formation of circular RNA [11, 23, 51]. If the intron has a unique inverted repeat sequence (such as Alu) [12, 52], after base pairing occurs, the splicing donor is brought close to the splicing acceptor, which promotes nucleophilic attack and splicing and can also promote the production of circRNA. However, the biochemical environment and regulatory factors required for the occurrence of circRNA are not yet clear. It is still worth noting that one gene can generate different circRNAs, which can be affected by the competition of RNA pairing across the flanking introns [3, 11].

### Function mechanisms of circRNAs

To date, studies using the application of emerging approaches have elucidated various regulatory mechanisms of circRNAs, which highlight many aspects of gene expression, DNA damage, RNA editing and immunity. We will focus on the representative epigenetic regulation of circRNAs (Fig. 1b–g), including circRNA-miRNA sponges, circRNA:DNA hybrids (circR-loops), guiding A-to-I editing, circRNA-protein interactions, and translation [16, 22, 27–29, 32].

A majority of studies have shown that circRNA can act as miRNA sponges in a manner similar to that of mRNA [22]. Circular RNA exists in the cytoplasm and has multiple miRNA binding sites. It can sponge miRNA to inhibit the regulatory function of miRNA. For example, miR-7 [53, 54] has been identified as a tumor-inducing factor or tumor suppressor in the process of tumorigenesis. Circular RNA (ciRS-7; also known as CDR1as) can specifically sponge miR-7, thereby inhibiting the function of miR-7 and upregulating the expression of IRS2, EGFR and other related genes [22, 55] (Fig. 1b). Another well-known epigenetic regulatory mechanism of circRNAs is their interaction with RNA-binding proteins [23, 56] (Fig. 1c). circRNA interactions with RBPs could function as protein antagonists or as inhibitors of protein activity [10, 57, 58]. For example, circ-Foxo3 interacts with cell cycle-related proteins (including p21 and p27), thereby blocking the role of these proteins in the cancer cell cycle [57]. CircPABPN1 binds to HuR, suppresses the interaction of HuR with PABPN1 mRNA and reduces its translation [58]. Besides, endogenous circRNAs tend to form 16–26 bp duplexes and interact with double-stranded RNA (dsRNA)-activated protein kinase (PKR), which blocks innate immunity [18, 40] (Fig. 1d). CircRNAs have an extensive ability to regulate cellular processes, which may explain the epigenetic differences between cells in the same organism.

In recent years, some emerging epigenetic regulatory mechanisms of circRNAs have been illuminated. DNA: RNA immunoprecipitation sequencing (DRIP-seq) data have also shown that circRNAs frequently form R-loop structures and tend to regulate DNA damage and genome instability [16, 59] (Fig. 1e). Some circRNAs can act as stable antisense RNAs to bind with RNAs to modulate RNA stability, structure, and activity [27, 60, 61] (Fig. 1f). For example, artificial antisense sequences in a circular RNA backbone can significantly reduce the proliferation of the SARS-CoV-2 virus [60]. Circular guide (g)RNAs were engineered to execute A-to-I editing on mRNAs by recruiting endogenous ADARs, which may realize the aim of treatment without disturbing genes [27].

### Translation potential of circRNAs

As mentioned above, circRNAs are a class of noncoding RNAs, but recent scientific research has shown that some circRNAs also have certain coding capabilities [32]. The 5' cap and 3' poly(A) tail are necessary structures for the linear translation of mRNA [25]. Unlike ordinary mRNA, circRNA lacks a similar translational molecular structure, but it can utilize the N6-adenosine methylation (m6A) modification or internal ribosome entry site (IRES) translation to promote the direct binding of the initiation factors to the cyclic RNA [25, 32, 62–64] (Fig. 1g). The translation of linear mRNA is initiated by the elF4E complex [65, 66]. First, elF4F binds to the 5' cap end of the mRNA, and then elF4G serves as a protein binding scaffold to assemble the initiation complex [66]. Then, the combination of elF3 and elF4G recruits ribosomes to the mRNA and initiates translation [66]. For circRNA, a special eIF4G protein (eIF4G2) directly recognizes IRES and initiates eIF4 complex assembly without eIF4E in a 5' cap-independent manner, providing circRNA with translation ability [29, 67]. m6A modification can also regulate the protein-coding potential of circRNAs [25, 68, 69]. For example, a high m6A methylation level was found in circZNF609, which promotes internal ribosome entry site (IRES)-activated protein coding [25, 68]. Yang et al. also examined the coding landscape of the human transcriptome and found that many circRNAs contain m^6^A motifs with translational potential and that high m^6^A levels in circRNAs have the ability to improve the efficiency of translation [25]. Interestingly, according to mass spectrometry, 50% of translatable endogenous circRNAs undergo rolling ring translation [32, 63, 67]. Given that circRNA lacks the general translational elements, a large number of products translated from circular RNAs are short in length and lower efficiency than that from mRNAs. Moreover, there are still issues that need to be further answered, such as which factors regulate the translation of circRNA, and what is the relationship between the translation product of circRNA and that of its corresponding linear transcript?

### CircRNA degradation

Due to the special structural characteristics of circRNA, it cannot be degraded by RNase H, which is conventionally used to eliminate linear RNA [15]. The specific degradation mechanism of circRNA is currently unclear. Several studies have found that miRNA can regulate the degradation of circRNA [22, 70]. For example, CDR1as can be degraded via sponging by miR-671 through Argonaute 2 (Ago2)-mediated degradation [55]. Circular intronic RNAs (ciRNAs) escape from DBR1 debranching of intron lariats and are cotranscriptionally produced from pre-mRNA splicing, but their turnover and mechanism of action have remained elusive [59]. Li et al. reported that RNase H1 degrades a subgroup of circular intronic RNAs (ciRNAs), which have high GC% and often form R-loops [16, 59] (Fig. 1h). For example, ci-ankrd52 facilitates R-loop formation, a process that allows the release of *ankrd52* pre-mRNA from R-loops by ci-ankrd52 replacement and subsequent ciRNA removal via RNase H1-mediated degradation [59]. This RNase H1/R-loop-dependent ciRNA degradation likely limits ciRNA accumulation and resolves R-loops at some GC-rich ciRNA-producing loci. In the autoimmune disease systemic lupus erythematosus (SLE), endogenous circRNAs bind to PKR via forming 16–26 bp imperfect RNA duplexes [18]. Upon viral infection, PKR is activated by phosphorylation in early cellular innate immune responses, resulting in the release of circRNAs and global degradation by RNase L [18] (Fig. 1i). This study suggests that the structure of circRNAs is important in innate immunity and its degradation. Studies have also found that m6A RNA modification can promote the recruitment of endonucleases to degrade circRNA [9, 19] (Fig. 1j).

In addition to intracellular degradation, circRNA can also be transported out of the cell in the form of exosomes and into body fluids [36, 71, 72]. However, the reason why cells form exosomes is still unclear. Is it merely a tool for the exchange of information between cells? Alternatively, it may reduce the toxicity caused by excessive accumulation of circRNA in the cell and actively transport circRNA out of the cell. The degradation of exosomes may release the circRNA outside; but there is no conclusive mechanism yet [73]. Although there are some endeavors to understand the mechanism of circRNA decay in certain contexts, further studies are still needed to fully understand the common circRNA degradation mechanisms under different physiological conditions.

### Principles and challenges for circRNA discovery and annotation

CircRNA constitutes a large amount of cell contents of unknown function [5, 9]. Accurate identification and annotation of novel emerging circRNAs are still urgently needed in this rapidly expanding research field. Recent advances in high-throughput RNA sequencing and related bioinformatics tools have accelerated research (Table 1). Since 2012, increasing numbers of bioinformatics tools have been developed to discover and annotate circRNAs. In 2013, find-circ became the first publicly available pipeline for identifying circRNAs from sequencing data [49]. Even today, many explorations of circRNAs still commence with RNA-seq data [74–77]. While RNase R-treated sequencing is considered easier and more accurate for circRNA detection, most circRNA detection tools can identify back-splice junction (BSJ) reads with high confidence from conventional RNA-seq datasets [2, 49, 78]. Nevertheless, achieving both sensitivity and specificity in circRNA discovery remains a challenge, particularly in the context of identifying and annotating novel emerging circRNAs.

**Table 1:** Bioinformatic tools for circRNAs discovery

Software	Seq type	Language	Latest update	Download link	Characteristic	Refs.
MapSplice	II	C++	2016	https://github.com/davidroberson/MapSplice2	/	[74]
PcircRNA_finder	II	Python, Perl	2016	http://ibi.zju.edu.cn/bioinplant/tools/manual.htm	Predict circRNAs in plants with frequently used circRNA detect tools	[75]
PredcircRNATool	II	Python	2016	https://sourceforge.net/projects/predicircrnatool/files	Identification of circular RNAs based on conformational and thermodynamic properties in the flanking introns	[108]
CircPro	II	Perl	2017	http://bis.zju.edu.cn/CircPro	Identify the protein-coding potential circRNAs	[198]
CIRI	II	Perl	2017	https://sourceforge.net/projects/ciri	De novo assemble novel circRNA with variable sequencing data	[82]
ACFS	II	Perl, Shell	2017	https://github.com/arthuryxt/acfs	Discovery and annotate circRNA from single-end RNA-seq	[91]
find_circ	II	Python	2017	https://github.com/marvin-jens/find_circ	De novo assemble novel circRNA transcripts and widely used in circbase	[49]
circseq-cup	II	Python	2017	https://github.com/bioinplant/circseq-cup	Identify full-length sequence of circRNAs	[207]
KNIFE	II	Python, Shell, Perl	2017	https://github.com/lindaszabo/KNIFE	Detect and quantify circRNAs from junctional alignments	[208]
PredcircRNA	II	Python	2017	https://github.com/xypan1232/PredcircRNA	Distinguish circRNA from other lncRNAs using multiple kernel learning	[76]
CPSS	II	PHP, Perl, R	2017	http://114.214.166.79/cpss2.0	For small RNA sequencing data analysis	[209]
miARma-seq	II	Perl, Python, R	2018	https://sourceforge.net/projects/miarma	Integration of mRNA, miRNA and circRNA analysis	[210]
CIRI-AS	II	Perl	2018	https://sourceforge.net/projects/ciri	Identify circRNA internal components and alternative splicing events de novo	[211]
hppRNA	II	Perl, R	2018	https://sourceforge.net/projects/hpprna	Analysis circRNA with different core-workflows from a large number of samples	[212]
segemehl	II	C + +	2018	http://www.bioinf.uni-leipzig.de/Software/segemehl	Detect back-splice reads and gene fusion	[83]
STARChip	II	Perl, Shell	2018	https://github.com/LosicLab/STARChip	Output the chimeric reads and discovery fusions circRNAs	[89]
UROBORUS	II	Perl	2018	https://github.com/WGLab/UROBORUS	Suggest detecting circRNAs with low expression levels in RNA-seq	[133]
WebCircRNA	II	Python	2018	https://rth.dk/resources/webcircrna/download	Using machine-learning based method to predict stem cell specific circRNAs	[213]
circRNA_finder	II	Perl, Awk, Shell	2019	https://github.com/orzechoj/circRNA_finder	/	[81]
CircRNAFisher	II	Perl	2019	https://github.com/duolinwang/CircRNAFisher	Identify circRNA de novo	[214]
PRAPI	III	Python	2019	https://pypi.org/project/prapi	One-stop solution of post-transcriptional regulation analysis for Iso-seq, suitable for third generation sequencing	[101]
CircRNAWrap	II	Shell, R	2019	https://github.com/liaoscience/circRNAwrap	Integrate multiple circRNA-detect tools to discovery confidence circRNAs	[85]
RAISE	II	Shell, Perl	2019	https://github.com/liaoscience/RAISE	Integrating detection, quantification and prediction of internal structure	[84]
DeepCirCode	II	Python, R	2019	https://github.com/BioDataLearning/DeepCirCode	Using machine-learning model to predict back-splice sites of circRNA	[77]
ROP	II	Shell, Python	2019	https://github.com/smangul1/rop	Discover the source of all reads with Python2, but it is no longer maintained	[215]
ACValidator	II	Python, Shell	2020	https://github.com/tgen/ACValidator	Assemble circRNA from pseudo-reference file	[216]
CircDBG	II	C + +	2020	https://github.com/lxwgcool/CircDBG	Detect circRNA by de Brujin graph	[217]
CircMarker	II	C + + . Java	2020	https://github.com/lxwgcool/CircMarker	/	[218]
AutoCirc	II	Perl	2020	https://github.com/chanzhou/AutoCirc	Identify back-splice junctions of potential circRNAs from RNA-seq de novo quickly	[24]
Pcirc	II	Python	2020	https://github.com/Lilab-SNNU/Pcirc	Identify plant circRNA with random forest methods	[110]
cirRNAPL	II	Java	2020	http://server.malab.cn/CirRNAPL	Identification of circRNAs based on extreme learning machine	[109]
circDeep	II	Python	2020	https://github.com/UofLBioinformatics/circDeep	Identification of circRNAs with deep learning	[111]
CLEAR	II	Python	2020	https://github.com/YangLab/CLEAR	Combine with ribo-seq & RNA-seq as input, and quantify the expression of circRNAs	[219]
NCLcomparator	II	Roff	2020	https://github.com/TreesLab/NCLcomparator	Detect circRNAs by combined several non-co-linear transcript	[220]
CIRCexplorer	II	Python	2021	https://github.com/YangLab/CIRCexplorer2	De novo assemble novel circRNA with supporting many common aligners	[13]
CIRI-full	II	Perl	2021	https://sourceforge.net/projects/ciri	Reconstruct and quantify full-length circular RNAs from RNA-seq data sets	[134]
CIRI-long	III	Perl	2021	https://sourceforge.net/projects/ciri	Identify circRNA from long-reads sequencing data	[102]
CIRIquant	II	Perl	2021	https://sourceforge.net/projects/ciri	Quantify circRNA expression from RNA-seq data	[221]
CirCompara2	II	Python, R	2021	https://github.com/egaffo/CirComPara2	Integrate multiple circRNA-detect tools to discovery confidence circRNAs	[86]
circAST	II	Python	2021	https://github.com/xiaofengsong/CircAST	Assemble full-length circRNAs and quantification using RNA-Seq data with the back-spliced events	[222]
DCC and CircTest	II	Python	2022	https://github.com/dieterich-lab/DCC	Detect and quantify circRNAs from chimeric reads	[78]
Ularcirc	II	R	2022	https://github.com/VCCRI/Ularcirc	Analysis and visualize the canonical and back-splice junctions, annotate circRNA with overlapping gene information	[80]
NCLscan	II	C + + , Python	2022	https://github.com/TreesLab/NCLscan	Identify both intragenic and intergenic non-co-linear transcript	[205]
circall	II	C + + , R	2022	https://github.com/datngu/Circall	Discovery circRNAs from paired-end RNA-seq	[223]
CYCLeR	II	R	2022	https://github.com/stiv1n/CYCLeR	Reconstruct and quantify circRNAs from RNA-seq datasets accurately	[224]
stackCirRNAPred	II	Python	2022	https://github.com/xwang1427/StackCirRNAPred	Identification of circRNAs based on stacking strategy	[107]
circtools	II	Python, R	2023	https://github.com/dieterich-lab/circtools	Integrate the cumbersome circRNA analysis process of analysis	[225]
circfull	III	Python	2023	https://github.com/yangence/circfull	Detect and quantify full-length circRNA isoforms from circFL-seq	[105]
isocirc	III	Python, R	2023	https://github.com/Xinglab/isocirc	Integrated pipeline to characterize full-length circRNA isoforms using rolling circle amplification	[104]

### Canonical BSJ-based circRNA identification

**Fig. 2:**
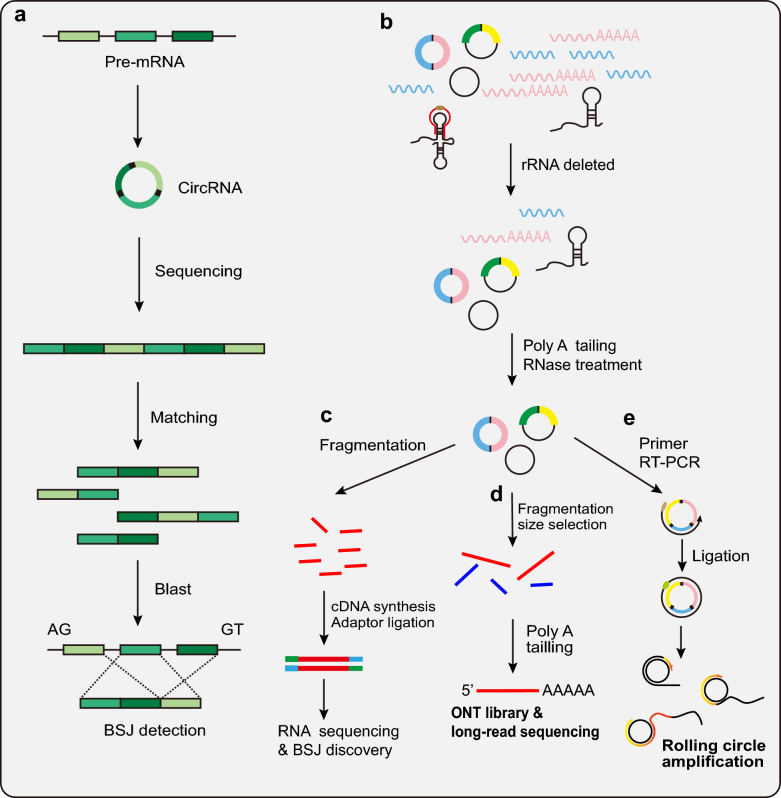
Workflow of BSJ-based circRNA identification. **a** The canonical workflow of circRNA identification tools that search for the specific BSJ sequences in the sequencing data and map to genome. **b** circRNA enriched by rRNA deleted and RNase R treatment before library. **c** circRNAs fragment into short reads and BSJ detecting in RNA sequencing. **d** After enrichment, the circRNA pool was nicked to generate large fragments, and then the obtained circRNA pool aligns with the ONT long-read sequencing protocol. **e** Discovery tools identify the full-length circRNA isoforms using rolling circle amplification followed by nanopore long-read sequencing

Many tools identify circRNAs by searching for specific BSJ sequences and performing different kinds of mapping (Fig. 2a). Most of the algorithms embedded in the tool are based on the segmentation of reads, while some other tools are based on predefined BSJ and circRNA flanking sequences. Examples include Find-circ [49], CIRI [79], CIRIexplorer [12, 13], Ularcirc [80], and circRNA-finder [81]. They all have their own merits or characteristics. Find_circ was the first circRNA prediction tool using the identification of back-spliced sequencing reads in RNA-Seq. CIRI, CIRI2 and CIRCexplorer2 [13, 79, 82] all scan through sequence data first to identify junction reads in backspliced exons, intron lariats, and alternative splicing sites and then implements multiple filtration strategies to remove false-positives. Other identification of BSJ reads is based on splicing, such as MapSplice [74] and segemehl [83]. MapSplice improves the quality and diversity of read alignments of a given splice to increase accuracy and can be used for both short (< 75 bp) and long reads (≥ 75 bp) to detect novel canonical as well as noncanonical splices [74].

Although circRNA library preparation of RNA-seq by rRNA deletion and RNase R treatment followed by many circRNA identification tools is a better method, there exist some RNase R-sensitive circRNAs, such as circ_CDR1as, which leads to the problem that these RNase R-sensitive circRNAs will be missing when only using RNase R-treated library preparation-based tools [15, 49, 82] (Fig. 2b, c). To improve circRNA identification efficiency and reduce the false-positive rate, some researchers integrate current prediction algorithms to make an ensemble tool (Table 1). For example, RAISE [84], CircRNAwrap [85], and PcircRNA_finder [75] that was used in the study of plants. Different integrated identification pipelines satisfy the different research purposes for users. Recently, Gaffo et al. developed CirComPara2 [86], which has been set to simultaneously use seven circRNA detection methods (integrated C2BW, C2SE, C2ST, C2TH, CIRI2 [82], DCC [78] and find_circ [49]) and identify the real circRNAs shared between at least two of these methods. The new trends of circRNA detection development are integrating variable tools because they can outperform single state-of-the-art circRNA identification tools and consistently achieve high recall rates without losing precision.

### Fusion circRNA identification

Previous studies have shown that fusion genes can transcribe into not only linear but also chimeric fusion circular RNAs (f-circRNAs), which are functional in gene expression regulation and implicated in malignant transformation [87–90]. Currently, even though it remains a challenge to identify fusion circRNAs owing to their general sparsity, low abundance in cells, heavy background noise in RNA-seq and perhaps imperfect computational methods, researchers have endeavored to develop bioinformatics approaches to systematically identify fusion transcripts, specifically detecting f-circRNAs in cancer cells (Table 1). ACFS has the ability to detect fusion events and recognize f-circRNAs from RNA-Seq data accurately [91]. However, f-circRNA detectors may suffer from a high false-positive rate and a significant increase in the computational burden owing to the detection algorithm performance. Identification of f-circRNAs requires detection of the BSJ site within the gene fusion events. STAR Chimeric Post (STARChip) is an open-source software based on the STAR aligner that can simplify filter high-quality chimeric alignments and improve f-circRNA identification to annotate f-circRNA in a rapid, efficient and scalable manner [89]. Cai et al. developed a comprehensive Python-based workflow called “Fcirc” to identify linear and circular RNA transcripts from known fusion events in RNA-Seq datasets [92]. It requires already known gene fusions as a reference to build the bipartite graph of gene pairs, which is different from fusion detection tools such as ChimeraScan [88], FusionCatcher [93], JAFFA [94], TrinityFusion [95] and STAR-Fusion [95]. Therefore, Fcirc can detect f-circRNAs from known fusion events with higher specificity, a lower false-positive rate and shorter computing times [92]. Usefully, Fcirc is an open-friendly comprehensive pipeline that can allow users to add their own fusion gene pairs of interest at their convenience and regularly update newly emerging fusion genes from common multiple databases (COSMIC, FusionCancer, ChimerDB, FARE-CAFE, and TicDB) [93, 96–99].

### circRNA identification using long-read sequencing data

The circRNA discovery tools above are mostly compatible with the reads of next-generation RNA-seq [2, 100]. Due to the short reads in RNA-seq, these alignment-based algorithms have difficulty distinguishing circular reads from the exonic regions that overlap the corresponding linear transcripts. In recent years, with emerging long-read sequencing technologies, including PacBio and Oxford Nanopore, reconstruction of transcript isoforms has become much easier [101–104]. Thus, the application of long-read sequencing technologies will lead to a novel generation of circRNA discovery tools that have the ability to achieve high-throughput detection of full-length circRNAs and improve sensitivity and specificity. circNick-LRS [103] (Fig. 2d) is the first reliable method to use long-read nanopore sequencing to detect circRNAs in both humans and mice.

Of note, due to circNick-LRS and circPanel-LRS eliminating the need for prior circRNA enrichment, a large number of nonconical splicing events in the global genome have been found to produce various types of circRNAs, including novel exons, intron retention and microexons. Both circFL-seq [105] and isoCirc [104] identify full-length circRNA isoforms using rolling circle amplification followed by nanopore long-read sequencing (Fig. 2e). Significantly, the low abundance circRNA reads could be enriched and identified using rolling circles and long-read sequencing. Zhao’s team developed an algorithm called the circRNA identifier using long-read sequencing data (CIRI-long) (Fig. 2e) to reconstruct the sequence of circRNAs [102, 106]. CIRI-long not only enables unbiased reconstruction of full-length circRNA sequences but also identifies mitochondria-derived circRNAs, transcriptional read-through circRNAs, and noncanonical AG/GT splicing circRNAs, which other methods to detect. Interestingly, CIRI-long identified a novel type of intronic self-ligated circRNA with a different incompletely characterized internal GT/AG splice signal rather than the flanking AG/GT signal in most exonic and intronic-exonic circRNAs [102]. With the development of sequencing technology, circRNA discovery tools provide insights into circRNA complexity that will further advance this rapidly expanding research field.

### circRNA identification using machine learning

Because the above methods always require RNA-seq data as input, circRNA signals with low abundance are usually missed [78, 100]. It is necessary for us to develop a novel tool to identify circRNAs at low levels. Machine learning algorithms establish some mapping rules based on the knowledge and characteristics of the real known circRNAs (Table 1). For example, PredcircRNA [76] and StackCirRNAPred [107] predict whether an unknown RNA sequence possibly comes from circRNA by some common reliable features, such as ALU repeats, structural motifs and sequence motifs [15, 76]. Other machine learning circRNA prediction tools based on the characteristics of nucleotide sequences are PredicircRNATool [108], DeepCirCode [77], CirRNAPL [109], PCirc [110], circDeep [111], etc. CirRNAPL is a user-friendly web server that extracts the structural features and pseudo-ribonucleic acid composition of circRNA to optimize the extreme learning machine based on the particle swarm optimization algorithm, which achieves identification accuracy in three public datasets [109]. Further improvements in the sensitivity and specificity of classifying circRNA from other lncRNAs can be found in circDeep, which is an end-to-end deep learning framework [111]. Considering the growing number of circRNA sequences and their splicing complexity, advanced parallel technology is highly recommended in circRNA discovery.

### Database for circRNA annotation and functional study

With the development of bioinformatic tools for circRNAs, an increasing number of public circRNA databases have emerged [20, 100, 112–114]. The most well-known and comprehensive database is circBase, which encompasses over 90,000 circRNAs along with their genomic coordinates, strands, annotations, and other relevant information [113]. These circRNA databases have become widely utilized in annotation pipelines, facilitating the research and analysis of circRNAs [100, 113]. Furthermore, several databases have been developed to gather diverse attributes of circRNAs beyond basic sequence information, offering unique features for research purposes [2, 64, 100, 115]. Notably, riboCIRC and TransCirc are comprehensive databases that specifically focus on potential translatable circRNAs [64, 116]. They provide predictions of circRNA-derived open reading frames (cORFs) and annotations of cORF-encoded peptides, supported by evidence of translation.

In recent years, the clinical significance of circRNAs has gained substantial attention, with increasing evidence showing their potential as clinical biomarkers and therapeutic targets [67, 114, 117, 118]. Specialized databases such as MiOncoCirc focus on providing information on the association between circRNAs and cancer [20]. Lnc2Cancer 3.0 has been updated to include circRNA-cancer associations and presents information on regulatory mechanisms, biological functions, and clinical applications of circRNAs in cancer [115]. Another comprehensive database, CircR2Disease v2.0 [119], provides experimentally validated relationships between circRNAs and various diseases. ExoRBase 2.0 concentrates on RNAs found in extracellular vesicles, encompassing circRNAs [120]. This database sheds light on the alterations of circRNAs in extracellular vesicles under both physiological and pathological conditions. At the same time, functional circRNA has emerged as a prominent research focus within the field of noncoding RNA. Several databases, including CircFunBase [112], deepBase [121], and circBank [122], provide valuable information on the interactions of circRNAs with various types of RNAs and proteins.

Despite progress in circRNA detection and annotation, the lack of standardized naming conventions remains a pressing issue in this field. The diverse naming methods used across different databases and articles have created a significant barrier for research, leading to information duplication and errors. Some databases use a 'circ_' prefix followed by a numeric ID or the parental gene symbol to name circRNAs [49, 113]. However, this inconsistent and arbitrary naming approach hampers the establishment of an integrated circRNA database. To address this issue, Chen et al. proposed a clear naming system for circRNAs. According to this system, a new circRNA can be named 'circ + ' followed by the parental gene name (separated by '::' in the case of fusion genes), the number of its exon, and 'RI' if it remains in an intron or 'S' if it exhibits different internal splicing patterns [50]. We strongly encourage researchers to embrace these clear naming rules to promote consistency and facilitate data integration.

### New insight into strategies to determine circRNA functions

Several methods have been developed to study the functions of circRNAs [9, 46]. We systematically summarized current strategies used to explore circRNAs, including ceRNA prediction [22], knockdown or out of functional circRNAs, overexpression of functional circRNAs [123–131], and circRNA-RBP prediction [132]. The advantages and disadvantages of these methods have also been discussed. Some new insights may help improve the strategies of circRNA research and applications of therapeutic potential.

### Strategies for circRNA detection

**Fig. 3:**
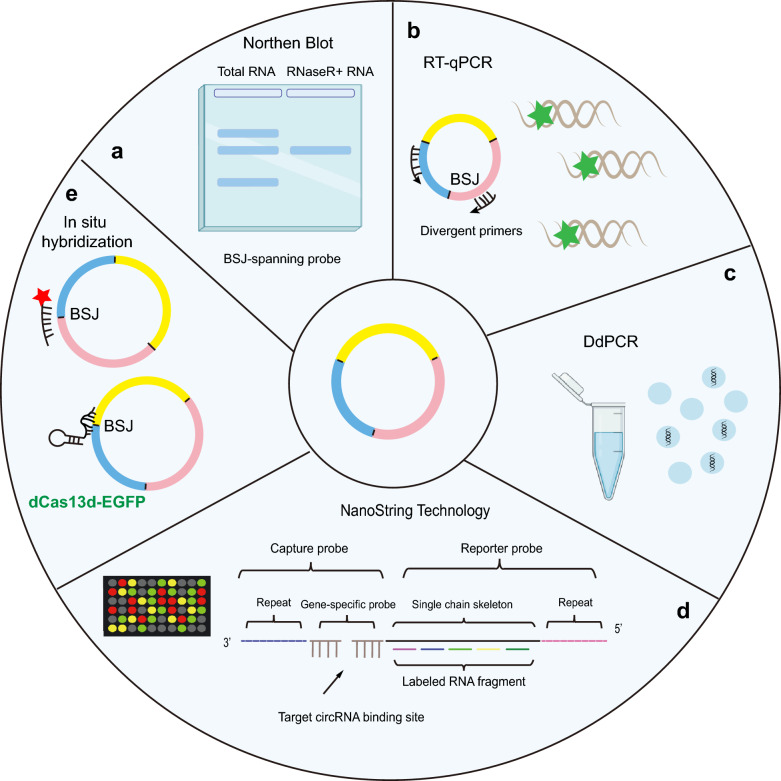
Strategies for circRNA detection. **a** Northern blot to identify and quantify circRNAs with a BSJ-spanning probe. **b** Quantification of circRNAs by reverse transcription (RT) and quantitative PCR (qPCR) assays. A pair of divergent primers are used. **c** Droplet digital PCR (DdPCR) is a novel technology determines the absolute quantification of a candidate circRNA using the ratio of positive to negative droplets. **d** NanoString Technology captured the BSJ flanking sequences by a biotinylated probe and a reporter probe loading fluorescent barcodes, followed by a high-resolution charge-coupled device (CCD) camera and digitization. **e** In situ hybridization used to visualize and quantify the interest circRNAs. There are two methods, which based on an oligonucleotide probe coupled to fluorescent dyes, and a sgRNA in a dCas13a-EGFP system

CircRNA sequencing of rRNA-depleted and RNase R-treated cells is the method used to discover novel circRNAs and was also used in all early circRNA profiling studies [20, 82, 133]. Based on the BSJ feature of circRNAs, candidate circRNAs were further identified and quantified. In recent years, many common detection techniques for various types of RNAs have also been applied in circRNA studies [78, 85, 105, 134]. Due to the lack of clarity regarding circRNA production or splicing, these detection methods have specific advantages and disadvantages (Fig. 3).

Northern blotting is the gold standard method for validating all kinds of RNAs, including circRNAs [9, 18, 123, 128]. Antisense probes are designed complementary to the sequences spanning the BSJ point in the circRNAs of interest, which are loaded on a denatured agarose gel containing formaldehyde, and hybridization is performed [18, 128] (Fig. 3a). This technique can precisely identify and quantify targeted circRNAs distinguished from linear RNAs transcribed from the same gene. However, the disadvantage of northern blotting is also obvious. This method requires a large amount of RNA, involves multiple steps, has a high background and often uses radioactively labeled probes [18]. This method generally requires many skills and is also time-consuming. Generally, candidate circRNAs are further validated and quantified by reverse transcription (RT) and quantitative PCR (qPCR) assays [2, 125, 135] (Fig. 3b). Although RT‒PCR is a timesaving and effective technique by means of a real-time PCR machine, the designed primer often cannot precisely distinguish the circular from the linear transcript during the fast PCR process with many copies of the amplified products [2]. The formation of concatemers by rolling circle amplification during the RT step is also a challenge that may hamper the accurate quantification of circRNAs.

Interestingly, droplet digital PCR (ddPCR) can overcome this shortcoming brought by RT‒qPCR [2, 33]. ddPCR is a novel technology that can determine the absolute quantification of a candidate circRNA using the ratio of positive to negative droplets, which exhibits a higher sensitivity even in plasma that has a very low amount of circRNA [33, 136] (Fig. 3c). However, the reagents for ddPCR assays are always expensive compared to other methods. If circRNAs can be quantified via high-throughput techniques, NanoString Technology is a good choice [4, 127] (Fig. 3d). The BSJ flanking sequences are captured by a biotinylated probe and a reporter probe loaded with fluorescent barcodes, and the circRNA-based barcodes on the reporter probes can finally be counted by a high-resolution charge-coupled device camera (CCD) and digitization. This enzyme-free technique also works well to detect paraffin-embedded RNA [4]. In situ hybridization (ISH) is another technique used to visualize and quantify circRNAs of interest [4, 125] (Fig. 3e). This technique designs an oligonucleotide probe, spanned to the BSJ site of circRNA, coupled to fluorescent dyes, to visualize a circRNA of interest in fixed and permeabilized cells using confocal microscopy. The value of fluorescent signals can reflect the quantity of circRNA to some extent. However, the ISH approach always requires the use of multiple probes covering the unique BSJ region, which may result in poor efficiency and a high false-positive rate. Interestingly, the dCas13a-EGFP system can be used to image and track specific circRNAs [137–139]. The special BSJ sequences could be a limit of guide RNA design in this approach.

### New insight into the knockdown/out of functional circRNAs

**Fig. 4:**
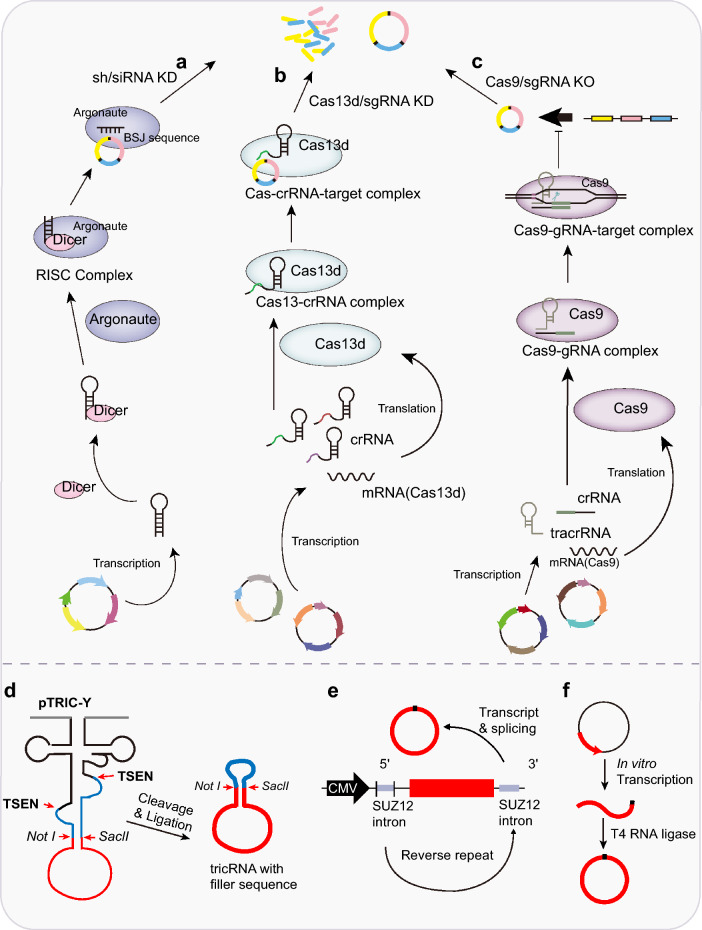
Strategies for knockdown/overexpression of circRNAs. **a** Lentivirus carrying shRNA according to the siRNA sequence to make the stable knockdown. sh/siRNA method executes the knockdown based on the complementary base pairing of seed sequences, which has 6–8 bases sponged to BSJ junction site. **b** CRISPR/Cas13d system degrades circRNAs that requires 28–30 nt long spacers and is intolerant to mismatches in spacers. **c** CRISPR/Cas9 system knocks out the special circRNA by deleting intronic complementary sequence neighboring the circularized exons. **d** Overexpression circRNA in a tRNA-derived intronic-circRNA system that followed a fluorescence-based RNA reporter allows to characterize the expression and localization visualization of circRNA. **e** Conformation of circRNA expression vector containing flanking introns from SUZ12 that splices to express circRNA without extra sequences. **f** In vitro synthesized RNA circles produced by T4 RNA ligase without extraneous fragments

Downregulating the expression of circRNAs is a popular strategy to explore their cellular functions [4, 5, 9]. Most circRNA knockdown methods are based on the complementary base pairing of seed sequences to BSJ junction sites, including siRNA, shRNA, ASO, or CRISPR/Cas series systems (Fig. 4a–c).

Introducing siRNA corresponding to circRNA specifically targeting BSJ into transfected cells is a convenient and effective method to inhibit the expression of circRNA in cancer cells [46, 125]. The cells can also be transfected with lentivirus carrying shRNA according to the siRNA sequence to achieve stable knockdown [46, 125] (Fig. 4a). However, the siRNA method executes the knockdown based on the complementary base pairing of seed sequences, which only has 6–8 bases sponged to the BSJ junction site, which may produce an off-target effect on the linear lncRNA or mRNA. The CRISPR/Cas13d system is a useful tool for efficiently degrading circRNAs and reducing false targeting [124, 129] (Fig. 4b). Efficient Cas13d knockdown requires 28–30 nt long spacers and is intolerant to mismatches in spacers [129, 140, 141]. For example, Li et al. constructed a CRISPR–RfxCas13d system and found that gRNA spacers with the BSJ in the center (–7 to 7 nucleotides spanning the BSJ site) exhibited high knockdown efficiencies without affecting linear cognate RNAs [124]. Because circular and linear RNA have distinct biogenesis efficiencies, conformations and turnover rates, RfxCas13d-based RNA interference specifically suppresses circular but not linear RNA [124]. Another advantage is that CRISPR/Cas13-based gRNA, which carry a spacer sequence specifically targeting and spanning the BSJ site within a relatively long sequence, should have the capability to distinguish between circular and linear RNAs and thereby reduce off-target effects on linear lncRNA or mRNA. The combination of lentiviral vehicle and CRISPR/Cas13d can help in investigating the function of circRNA specificity in a xenotransplantation model and drug sensitivity screening.

In recent years, CRISPR/Cas9, which is a highly specific and efficient tool to edit the genome, has also been used in circRNA knockout [123, 142]. In general, the CRISPR/Cas9 system knocks out special circRNAs by deleting intronic complementary sequences neighboring circularized exons in circRNA biogenesis [5, 46, 143–145] (Fig. 4c). For example, sgRNA specifically targeting the inverted complementary sequence in the intron of GCN1L1 can knock circGCN1L1 out but not disturb the corresponding linear mRNA [145]. Similarly, CRISPR/Cas9 removal of the downstream inverted repeat *ALU* element can prevent circHIPK3 formation [144]. However, due to the complexity of circRNA biogenesis, it is difficult to determine which intronic sequences are targeted by sgRNAs in the CRISPR/Cas9 system. Apart from targeting intronic sequences, another challenge of circRNA knockout using the CRISPR/Cas9 system is that many circRNAs are produced from alternative splicing between exons and introns in the genome. Alternative splicing-based circRNA cannot directly target the sequence by sgRNAs, which may interfere with linear mRNA production [146].

Therefore, it is still necessary to gain insight into circRNA knockdown-based strategies, which should be considered with many different factors involved in circRNA production.

### Overexpression of functional circRNAs

Several methods based on chemical synthesis and enzymatic ligation have been used to generate circRNAs in vitro; however, circRNA production in vivo has only recently been delineated [47, 128, 147, 148]. There is a circRNA-expressing vector that splices intron-containing tRNAs to produce circRNAs in cells [47, 148] (Fig. 4d). Construction of the tRNA-derived intronic-circRNA with a fluorescence-based RNA reporter allows us to characterize the expression of and visually localize circRNA. Because tRNA is constitutively expressed in all cells, tRNA-derived intronic circRNAs are theoretically expressed at high-copy and stable levels [47, 148]. Due to the feature of tRNA biogenesis by the processivity of pol III, this method have a circRNA size limitation (generally < 250 nt) [47]. Another in vivo circularized RNA was generated by the Group I intron of the phage T4 thymidylate synthase (td) gene transfected into cultured mammalian cells [62, 149]. However, both tRNA- and td gene-based RNA circles induced some extra sequences that tended to form 16–26 bp imperfect dsRNA regions, which generally activated remarkable immune responses via recognition by the pattern recognition receptor retinoic-acid-inducible gene I (RIG-I) or PKR [62, 128, 149]. We previously constructed a universal circRNA expression vector containing flanking introns from SUZ12 that ensured correct splicing to express circRNA without extra sequences [125] (Fig. 4e). We added a sequence that is the reverse complement repeat of the first 100 bp of the 5’ intron component into the vector following the 3’ intron to promote the interaction between the flanking introns, facilitating circRNA production. For example, the sequence of exons 8–9 of MYBL2 was inserted into the vector, and circMYBL2 was highly expressed, i.e., approximately 100-fold, in 293 T cells [125].

Considering the complexity of circRNA biogenesis, suitable strategies are needed for studying the different structural and functional features of circular RNA occurring in cells [5].

The replacement of stronger enhancers including ICSs, Alu elements, other RNA pairing structures and adding BSJ associated RBPs may be strategies to improve circRNA overexpression [5, 12, 150]. In contrast, Chen’s laboratory introduced in vitro synthesized RNA circles produced by T4 RNA ligase without extraneous fragments that present minimized immunogenicity, suggesting a useful method for the future synthesis of circular RNAs [128] (Fig. 4f).

### ceRNA prediction

**Fig. 5:**
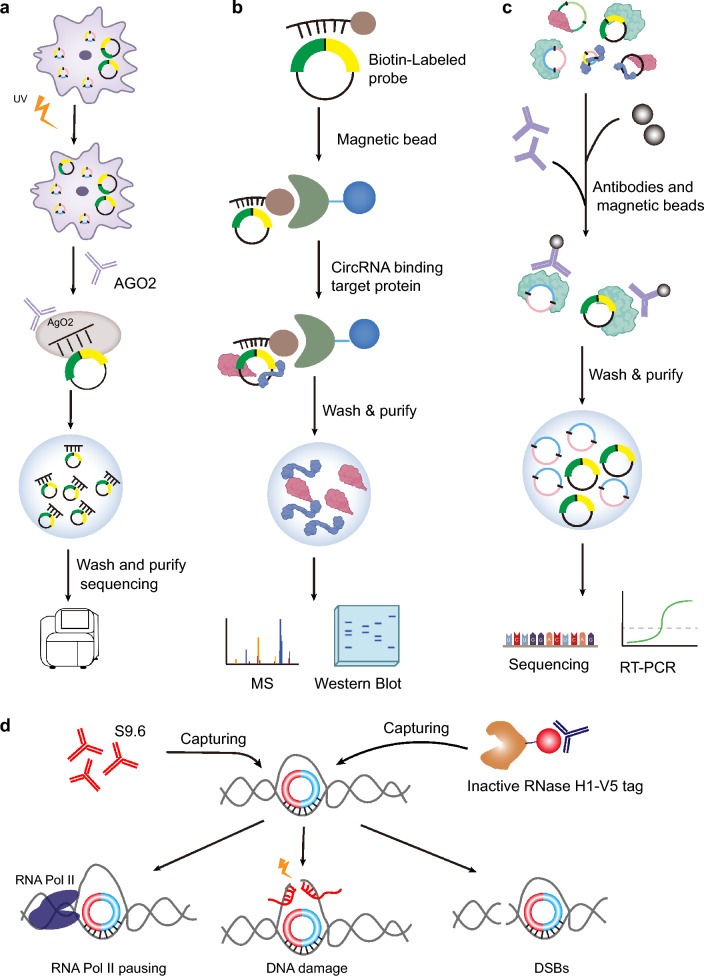
Methods to explore the possible mechanisms of circRNAs. **a** AGO2 CLIP-seq to predict the communication between miRNA and targeted circRNA. **b**,** c** circRNA-RBP interactions is mainly through RNA pull-down assay (**b**) or RNA immunoprecipitation (RIP) for experimental analysis (**c**). **b** In the RNA pull-down assay, a biotin-labeled probe recognized the BSJ of circRNA and then captured by biotin coupled magnetic beads. Finally, mass spectrum (MS) and western blot analysis to determinate the circRNA binding proteins. **c** In RIP-seq assay, the candidate RBP was first binds to the magnetic beads via antibody, and then the interacted circRNAs were analyzed by sequencing and RT-PCR. **d** circR-loops are identified by immunoprecipitation with the R loop-specific S9.6 antibody or catalytically inactive human RNase H1. Discovery the circR-loops in DRIP-seq data. circR-loops regulate diversity types of biological process, including transcriptional pausing, DNA damage, and double-strand DNA breaks (DSBs)

When circRNAs enter the cytoplasm, some of them become competitive endogenous RNAs (ceRNAs) [22, 100]. CircRNA can bind miRNA to prevent it from binding to target genes and changing the regulatory ability of target gene mRNA. Bioinformatics algorithms can be used to predict whether circRNAs have matching miRNAs [151–153] (Fig. 5a). The AGO2 protein was identified by analyzing the experimental data for CLIP-seq and functional genomic annotations, and the communication between miRNA and targeted circRNA was predicted after analysis and processing [151].

### circRNA-RBP prediction

Although circRNA-miRNA sponging is the most well-known function, increasing evidence has also shown that circRNAs can interact with RBPs to exert widespread regulatory effects [56, 132]. For example, circPABPN1 can bind to HuR and prevent HuR from binding to PABPN1 mRNA, thereby reducing the translation of PABPN1 [154]. Some databases have summarized the interactions between circRNAs and RBPs. For example, CircInteractome provides miRNA and RBP binding sites on circRNA [132]. starBase also concentrated and systematically identified RNA‒RNA and protein‒RNA interaction networks [151].

To date, experimental research on circRNA-RBP interactions has mainly been conducted through RNA pulldown assays or RNA immunoprecipitation (RIP) for experimental analysis [56, 155] (Fig. 5b and c). Although these methods have been popularly used in many important discoveries, they still face many difficulties such as high costs, large tasks, and time consumption. Therefore, some programs that can predict the interaction of circRNA and RBP have been developed to compensate for the defects of classic experiments [56, 156]. Wang's team used matrix factorization and neural networks (MFNNs) to construct a prediction framework based only on interaction matrices, which has a high prediction accuracy and is an effective prediction method [156]. CirRBP, a stacked operation ensemble deep learning model, can fuse binding sites from multiple databases via a localization algorithm and compensates for the defect that most previous prediction methods only identify circRNA-RBP binding sites based on a single data resource [56]. However, CirRBP cannot provide accurate binding sites but only provides probability values of sequence fragments. Then, CirRBP was developed into an open-source web application called CRWS, which can allow users to change the codes in their own needs. CRWS is a useful online tool to use multi-source data to train models and predict precise binding sites [56]. Therefore, highly efficient and convenient circRNA-RBP prediction strategies will undoubtedly be useful for the study of circRNA functions.

### circR-loops: circRNA:DNA hybrids

R-loops are widespread structures that are often formed co-transcriptionally [59, 157–159]. The genome-wide R-loop signature was generally identified by immunoprecipitation with the R loop-specific S9.6 antibody or catalytically inactive human RNase H1 (dRNH1) coupled with high-throughput sequencing of the resident DNA and RNA [59, 158, 160]. Apart from nascent mRNAs, DRIP-seq data have also shown that lncRNAs and circRNAs frequently form R-loop structures [17, 161] (Fig. 5d). These pervasive formations of circR-loops regulate diverse types of biological processes, including gene expression and DNA damage in cells [16, 17, 161–163]. For example, circSEP3 can form an R-loop by binding strongly to its cognate DNA locus, leading to SEPALLATA3 transcriptional pausing and coinciding with alternative splicing [163]. Overexpression of circSMARCA5 can generate a circR-loop at its parent gene locus, which results in transcriptional pausing at exon 15 of SMARCA5 and is sufficient to improve sensitivity to cytotoxic drugs in breast cancer [162]. Interestingly, a recent study showed that a set of circRNAs are enriched within the breakpoint cluster region (bcr) of MLL and can form circR-loops at their cognate loci [17]. These circR-loops promote transcriptional pausing, proteasome inhibition, chromatin reorganization, and double-strand DNA breaks (DSBs). Overexpressing circMLL (9,10) can trigger the de novo generation of clinically relevant chromosomal translocations mimicking the *MLL* recombinome in mouse leukemia xenograft models [17]. These studies suggest that nuclear circRNAs may form circR-loops and play both physiological and pathological roles in cells. Abnormalities in circRNA export from the nucleus can lead to diseases. Chen et al. identified that conserved exportin 4 (XPO4) can modulate circRNA nuclear export [16]. They observed that knockdown of XPO4 can improve circRNA nuclear retention, circR loop formation and DNA damage [16].

Recent studies may suggest that many circRNAs in circR-loops regulate the cognate DNA locus or mRNA transcription in a *cis* manner [16, 17]. It is still unclear whether these circRNAs in circR-loops can play roles in *trans*. There is still an interesting question that whether circR-loops interact with special RBPs to mediate chromatin marks, chromatin accessibility or active chromatin landscape.

### New insights into biomedical application of cancer-related circRNA

Because circRNA has tissue- and cancer-specific expression and stability in body fluids, it can be used as a rapid, accurate, and noninvasive biomarker for early diagnosis and prognosis [20, 37, 114, 130, 164]. Several circRNAs are reported to play important roles in tumorigenesis and progression, as well as in chemotherapeutic resistance, and are potential promising targets in cancer treatment [66, 115, 130].

### CircRNA is a promising biomarker in cancer

Cancer cells present aberrant expression of circRNAs, which are usually related to some clinical characteristics, such as tumor type, tumor size, histological grade, tumor invasion and metastasis (Table 2). For example, in non-small cell lung cancer, low expression of hsa_circ_0001073 may distinguish adenocarcinoma from squamous cell carcinoma [165]. In breast cancer, circRNA expression profiles may distinguish between estrogen receptor-positive, HER2-positive, and triple-negative breast cancer [166]. In tissue samples, the upregulation of hsa_circ_0003823, circPUM1, circCYP24A1, and circCNOT6L presented diagnostic performance with considerable sensitivity and specificity values, which exhibited relatively higher recurrence of esophageal squamous cell carcinoma (ESCC) [167–170]. In the plasma samples, Hu et al., found that highly concentration of plasma circGSK3β and CEA can indicate the recurrence/metastasis of ESCC [171]. CircRNA also showed the ability to distinguish different nontumor diseases [172]. The hsa_circRNA_0001599 was highly expressed in large-artery atherosclerosis (LAA)-stroke patients, revealing its potential as a biomarker of LAA-stroke diagnosis [172]. The plasma concentration of CircBRAP can be a predictor of preeclampsia [173]. CircRNA can be quite stable in biological fluids, and detection of circulating circRNA may be an excellent noninvasive biopsy that is likely to become a new method for cancer detection in the future.

**Table 2:** Cancer-related circRNAs

Cancer	Name	Up/down	Characteristic	Refs.
*Hematologic malignancies*				
AML	Circ_0009910	Up	Silencing Circ_0009910 can significantly inhibit proliferation, sphere formation and promote apoptosis	[226]
AML	Circ-SFMBT2	Up	Silencing Circ-SFMBT2 can inhibit the proliferation, migration, invasion and glycolysis of AML cells and induce apoptosis	[227]
AML	circ_0040823	Down	Overexpression of circ_0040823 inhibited the proliferation of AML cells and induced apoptosis and cell cycle arrest	[184]
AML	hsa_circ_0079480	Up	Associated with overall survival and relapse-free survival of AML	[228]
AML	circ_0004277	Down	Overexpression of circ 0004277 inhibited the migration and invasion of AML cells	[183]
ALL	Circ_0000745	Up	Knockdown of Circ_0000745 inhibits cell cycle progression and glycolysis, and induces apoptosis and iron death	[229]
ALL	circ_0008012	Up	related to proliferation and apoptosis of ALL cells	[230]
CLL	circ-CBFB	Up	Knockdown of circ-CBFB inhibited the proliferation of CLL cells, stopped the cell cycle and induced apoptosis	[231]
CLL	hsa_circ_0132266	Down	Inhibition of CLL cell apoptosis and impaired proliferation	[232]
CLL	Hsa_circ_0064574	Up	highly expressed in the plasma of CLL patients	[233]
CLL	circZNF91	Up	Silencing circZNF91 can inhibit CLL cell proliferation, induce apoptosis and block cell cycle	[234]
CML	Hsa_circ_0058493	Up	Increase the resistance of CML cells to imatinib	[235]
CML	circ_0080145	Up	Increase the resistance of CML cells to imatinib	[236]
CML	circ_0051886	Up	Increase the resistance of CML cells to imatinib	[236]
MM	Circ_0000190	Down	Inhibiting the viability, proliferation and inducing apoptosis	[237]
MM	hsa_circ_0007841	Up	Associated with drug resistance and chromosome aberration	[38]
MM	circITCH	Down	Related to the resistance of MM cells to bortezomib (BTZ)	[238]
*Digestive system malignancy*				
CRC	Hsa_circ_0082182	Up	Associated with tumor proliferation and lymph node metastasis	[239]
CRC	Hsa_circ_0000370	Up	Associated with tumor proliferation and lymph node metastasis	[239]
CRC	hsa_circ_0004585	Up	Positively correlated with tumor size	[240]
CRC	hsa_circ_0000567	Down	Negatively correlated with tumor size, lymph node metastasis, remote metastasis, and TNM staging	[241]
CRC	hsa_circ_0004771	Up	Upregulated in tumor cell-derived plasma exosomes	[242]
HCC	circIPO11	Up	Drives self-renewal of liver cancer	[123]
HCC	hsa_circ_0000798	Up	High expression in liver cancer tissues was negatively correlated with the overall survival cycle of patients	[243]
HCC	hsa_circ_0027089	Up	Distinguishing cirrhosis	[244]
HCC	hsa_circ_0058124	Up	Associated with invasive characteristics, also regulates the resistance of liver cancer cells to sorafenib	[245]
HCC	hsa_circSMARCA5	Down	Related to proliferation, invasion and metastasis	[246]
HCC	hsa_circ_0068669	Down	Related to tumor microvascular invasion and TNM staging	[247]
HCC	hsa_circ_0028502	Down	associated with lymph node metastasis and TNM stage	[248]
HCC	hsa_circ_0076251	Down	Associated with Barcelona Clinic Liver Cancer (BCLC) stage	[248]
HCC	circUBAP2	Up	Negatively correlated with aggressive clinical characteristics	[249]
HCC	circRNA-YBX1	Down	Mediate phase separation suppresses the metastasis	
GC	circNRIP1		Inhibit the growth of gastric cancer	[179]
GC	hsa_circ_0003159	Down	Negative correlation between tumor metastasis and TNM stage	[250]
GC	hsa_circ_0000096	Down	Affects the growth and migration of GC cells	[251]
GC	hsa_circ_002059	Down	Associated with distal metastasis of tumor cells and TNM staging	[252]
GC	hsa_circ_0000190	Down	Related to tumor diameter, lymphoid metastasis, distal metastasis and TNM stage	[253]
GC	hsa_circ_0000181	Down	Associated with tumor diameter, lymphoid metastasis	[254]
GC	hsa_circ_0000467	Up	Closely related to TNM staging	[255]
GC	hsa_circ_0001895	Down	Down-regulated in GC tissue and precancerous stage of GC	[256]
GC	hsa_circ_0017728	Up	Associated with short overall survival, poor pathological differentiation, higher TNM stage and lymph node metastasis	[257]
GC	circPDIA4	Up	Accelerate the invasion of cancer cells in vitro, promote the progression of GC and indicate poor prognosis	[258]
BC	Hsa_circ_0001136	Up	Associated with tumor grade, tumor stage, lymph node invasion and distal metastasis	[259]
BC	hsa_circ_0137439	Up	Related to tumor grade, tumor stage, lymph node invasion, also can distinguish between MIBC and NMIBC	[260]
BC	hsa_circ_0001361	Up	Promoted the invasion and metastasis of bladder cancer cells and was positively correlated with pathological grade	[261]
BC	circSLC8A1	Down	Overexpression inhibits the migration, invasion and proliferation of tumor cells	[262]
PC	circANAPC7	Down	Inhibits Tumor Growth and Muscle Wasting	[180]
PC	Circ-MBOAT2	Up	Regulates cell proliferation, migration, invasion and glutamine catabolism	[181]
PC	circRNA IARS	Up	Positively correlated with hepatic metastasis, vascular infiltration and TNM stage of pancreatic ductal adenocarcinoma (PDAC), and negatively correlated with postoperative survival time	[263]
PC	hsa_circRNA_001859	Down	Inhibit the proliferation, invasion and EMT of pancreatic cancer	[264]
OSCC	Hsa_circ_0001971	Up	Related to TNM stage of tumor	[265]
OSCC	Hsa_circ_0001874	Up	Related to tumor grade and TNM stage	[265]
OSCC	Hsa_circ_0003829	Down	Negatively correlated with lymph node metastasis and TNM stage	[266]
OSCC	Circ_0109291	Up	Silencing circ_0109291 can improve tumor sensitivity to DDP	[267]
ESCC	Hsa_circ_0003823	Up	Promotes the Tumor Progression, Metastasis and Apatinib Resistance	[167]
ESCC	circPUM1	Up	Regulates oxidative phosphorylation	[168]
ESCC	circCYP24A1	Up	Facilitates esophageal squamous cell carcinoma progression	[169]
ESCC	circCNOT6L	Up	Regulates cell development	[170]
ESCC	circGSK3β	Up	Promotes metastasis	[171]
EC	circ-VIM	Up	Silencing circ-VIM in vitro can inhibit immune escape and multiple carcinogenic activities of EC cells, as well as inhibit internal xenograft growth and lung metastasis	[182]
*Lung cancer*				
LC	Hsa_circ_0001715	Up	Related to TNM stage and distant metastasis of lung adenocarcinoma, and inversely proportional to overall survival	[268]
LC	Hsa_circ_0005962	Up	Promote the proliferation of lung adenocarcinoma cells (LUAD)	[269]
LC	Hsa_circ_0086414	Down	Plasma hsa_circ_0086414 was related to EGFR mutations	[269]
LC	Hsa_circ_002178	Up	Promotes the expression of PDL1/PD1 in lung adenocarcinoma cells and is also present in exosomes	[270]
LC	Hsa_circ_0037515	Down	Significantly down-regulated in non-small cell lung cancer (NSCLC)	[271]
LC	Hsa_circ_0037516	Down	significantly down-regulated in non-small cell lung cancer	[271]
LC	hsa_circ_0001073	Down	Indicates the lung adenocarcinoma (LUAD) subtype in non-small cell lung cancer	[165]
LC	hsa_circ_0001495	Up	Indicates the squamous cell carcinoma (LUSC) subtype in non-small cell lung cancer	[165]
*Others*				
RC	circHIAT1	Down	Overexpression inhibits the malignant progression of clear cell renal cell carcinoma	[272]
RC	hsa_circ_001895	Up	Promotes ccRCC cell proliferation, invasion and migration and is associated with poor prognosis	[273]
GM	circRNA-104718	Up	Indicates a poor prognosis and promotes invasion and migration of tumor cells	[274]
GM	circ-GLIS3	Up	Related to the resistance of temozolomide (TMZ) and promotes the proliferation, invasion and migration of glioma cells	[275]
GM	Circ_0047688	Up	Promote malignant behavior of glioma cells	[276]
GM	Circ_0001982	Up	Promote the proliferation, migration and invasion of glioma cells	[277]
GM	has-circ-0072688	Up	Promote the proliferation of glioblastoma and inhibit apoptosis	[278]
GM	hsa_circ_0030018	Up	Promote proliferation and inhibit apoptosis of glioma cells	[279]
Breast cancer	hsa_circ_0008673	Up	Related to tumor size and distal metastasis	[280]
Breast cancer	Circ-LARP4	Down	High expression indicates good prognosis and is negatively correlated with tumor size	[175]
Breast cancer	circRNA-CREIT	Down	Increases drug resistance in triple negative breast cancer (TNBC) and is associated with poor prognosis	[40]
OC	circBNC2	Down	associated with advanced cancer and lymph node metastasis in epithelial ovarian cancer (EOC)	[281]
TC	Hsa_circ_0137287	Down	related to tumor size, lymph node metastasis and TNM stage	[282]
CC	Circ_0000745	Up	Knockdown Circ_0000745 inhibited proliferation, migration, invasion and glycolysis of cervical cancer cells	[283]

AML: Acute Myelocytic Leukemia; ALL: Acute Lymphocytic Leukemia; CLL: Chronic Lymphocytic Leukemia; CML: Chronic Myeloid Leukemia; MM: Multiple Myeloma; CRC: Colorectal Carcinoma; HCC: Hepatocellular Carcinoma; GC: Gastric Carcinoma; BC: Bladder Cancer; PC: Pancreatic Cancer; OSCC: Oral Squamous Cell Carcinoma; ESCC: Esophageal Squamous Cell Carcinoma; EC: Esophagus Cancer; RC: Renal Carcinoma; GM: Glioma Malignancy; OC: Ovarian Cancer; TC: Thyroid Cancer; CC: Cervical Cancer; LC: Lung Cancer

CircRNAs can not only distinguish different tumor subtypes but also indicate different prognostic levels in the body [130, 174, 175]. For example, CIRS-7 is associated with poor prognosis in most cancers [174]; circUBAP2 has also been identified as an oncogenic factor associated with poor prognosis [174], while circLARP4 is a tumor suppressor associated with good prognosis in several cancers [176]. circRNA-CREIT was also recently found to be abnormally downregulated in doxorubicin-resistant triple-negative breast cancer (TNBC) cells and associated with poor prognosis [40].

### CircRNAs are promising therapeutic targets

In recent years, numerous dysregulated circRNAs have been found to affect the proliferation, apoptosis, metastasis, DNA damage and other life activities of cancer cells [3, 10, 99, 130]. Therefore, similar to miRNAs and lncRNAs, circRNAs can also be used as therapeutic targets for cancer treatment [54, 130, 177, 178] (Table 2). For example, intratumoral injection of circNRIP1 siRNA could significantly inhibit the growth of gastric cancer in PDX mouse models, suggesting that oncogenic circNRIP1 may be a promising target for gastric cancer treatment [179]. Antisense oligonucleotides (ASOs) against circIPO11 combined with the TOP1 inhibitor camptothecin (CPT) exert synergistic effects and can significantly suppress liver cell self-renewal and HCC propagation [123]. The knockdown of circMYBL2 in vitro and in vivo by siRNA and shRNA significantly inhibited the FLT3-ITD protein level and inhibited the proliferation of FLT3-ITD AML cells but had no effect on normal cells [125]. circIPO11 knockout using CRISPR/Cas9 technology suppresses the progression of chemically induced liver cancer development [123]. Notably, several circRNAs act as suppressors in cancer progression, indicating their antitumor effects [154, 180–184]. circANAPC7, newly discovered tumor suppressors, can significantly inhibit tumor growth and muscle atrophy in pancreatic cancer [180]. In vivo delivery of these kinds of tumor suppressor circRNAs may be a promising approach for anticancer therapy.

## CircRNA regulates therapy resistance and targeted drug development

**Fig.6:**
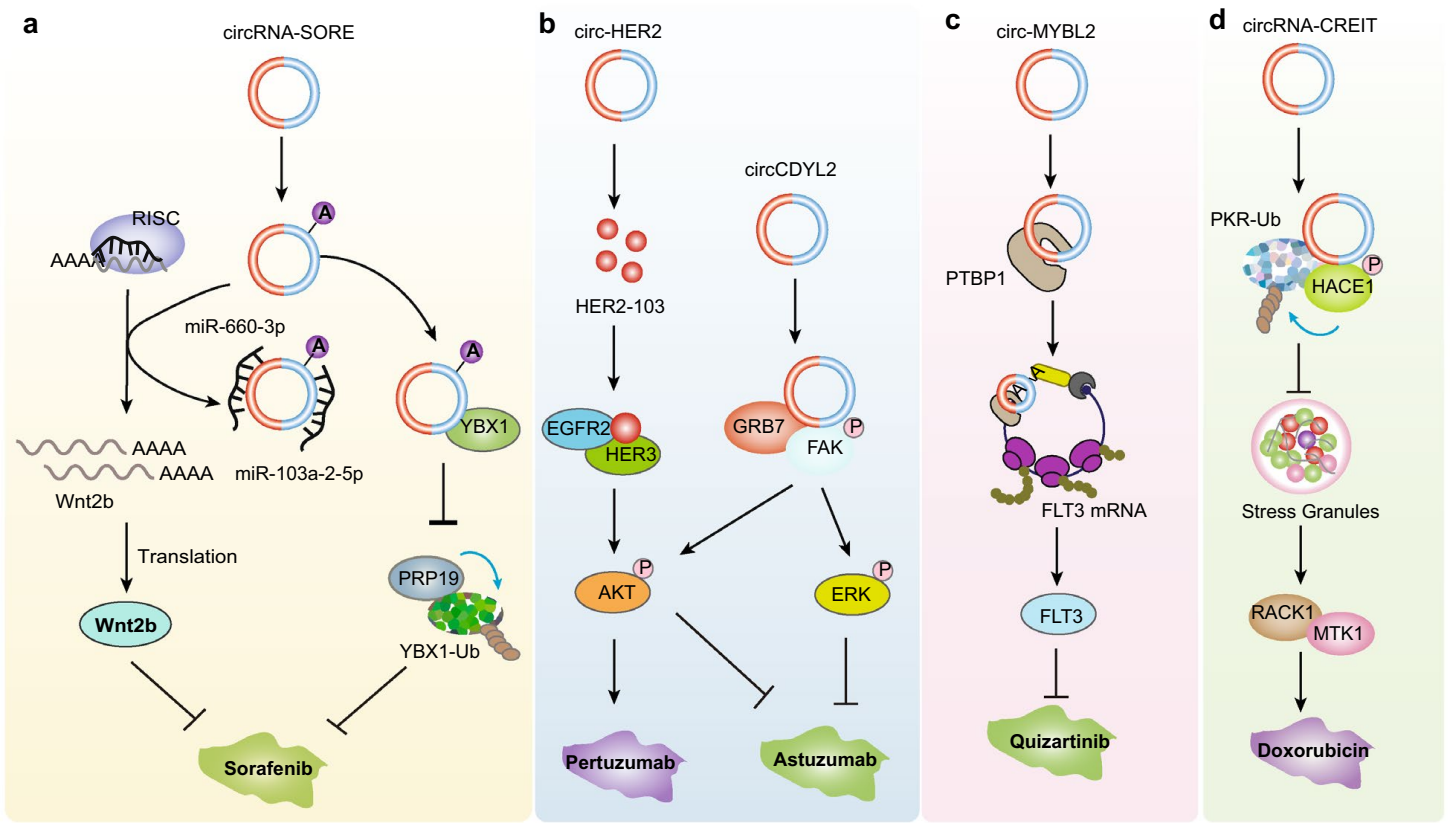
CircRNA regulates therapy resistance. **a** circRNA-SORE acts as ceRNA to miR-103a-2-5p and miR-660-3p, and competitively activates Wnt2b translation to promote sorafenib resistance in HCC; it also binds to YBX1 and blocks PRP19-mediated YBX1 degradation to regulate sorafenib resistance. **b** circCDYL2 enhances the interaction between GRB7 and FAK, thereby activates AKT and ERK1/2 signaling pathways to promote trastuzumab resistance in breast cancer. Circ-HER2 can also encode a small protein HER2-103, which promotes homo/hetero dimerization of epidermal growth factor receptor (EGFR)/HER3, and actives AKT phosphorylation, which endows the sensitivity to Pertuzumab in triple-negative breast cancer. **c** circMYBL2 regulates FLT3-ITD translation by binding of PTBP1 to FLT3 messenger RNA in quizartinib-resistant FLT3-ITD AML. **d** circRNA-CREIT facilitates the interaction between PKR and the E3 ligase HACE1 and promoted proteasomal degradation of PKR protein, thereby attenuating the stress granules (SGs) assembly to activate the RACK1/MTK1 apoptosis signaling pathway and overcome doxorubicin resistance

In the current clinical treatment of cancer, various chemotherapeutic drugs have been developed to inhibit the growth of cancer cells and have achieved good clinical effects [185–187]. However, with the prolonged time of medication at any time, the drug resistance of cancer cells gradually increases, resulting in the gradual weakening of the therapeutic effect, which is a major problem that has to be solved in clinical treatment [187, 188]. Recent studies show that circRNAs play a role in the resistance of cancer cells to anticancer agents [33, 189, 190]. They found that circRNA-SORE (also known as circRNA_104,797 and circ_0087293) was upregulated in sorafenib-resistant HCC cells, acting as ceRNA to isolate miR-103a-2-5p and miR-660-3p and competitively activate the Wnt/β-catenin pathway to promote sorafenib resistance [191] (Fig. 6a). Interestingly, this team also reported that circRNA-SORE binds YBX1 and blocks PRP19-mediated YBX1 degradation. They found that silencing circRNA-SORE by injection of siRNA in vivo could substantially overcome sorafenib resistance [41] (Fig. 6a). CircVMP1 could upregulate the expression of methyltransferase 3, N6-adenosine-methyltransferase complex catalytic subunit (METTL3) and SOX2 by acting as a sponge of miR-524-5p, thereby promoting the progression of NSCLC and cisplatin (DDP) resistance [192]. These studies put forward a new idea for solving chemotherapeutic drug resistance by knocking down specific circRNAs to inhibit their function of promoting drug resistance.

CircRNAs can also interact with oncoproteins to help cancer cells establish drug resistance [33, 189, 193, 194]. For example, circCDYL2 enhances the interaction between GRB7 and FAK by inhibiting the ubiquitination degradation of GRB7, thereby maintaining the activation of downstream AKT and ERK1/2 signaling pathways and leading to trastuzumab resistance in breast cancer [193] (Fig. 6b). Circ-HER2 encodes the small protein HER2-103, which promotes homo/heterodimerization of epidermal growth factor receptor (EGFR)/HER3 and activates AKT phosphorylation and malignant phenotypes [194]. Pertuzumab inhibits the tumorigenicity of circ-HER2/HER2-103-expressing TNBC cells but not circ-HER2/HER2-103-negative TNBC cells in vivo [194]. These studies suggest that both knockdown of circCDYL2 and overexpression of circ-HER2/HER2-103 together can improve the outcome of drug therapy targeting HER2 signaling in TNBC. We previously also showed that circMYBL2 is more highly expressed in AML patients with FLT3-ITD mutations [125] (Fig. 6c). Relapse of FLT3_ITD AML has been observed due to acquired resistance with secondary mutations in FLT3. shRNA-mediated circMYBL2 knockdown specifically inhibited FLT3-ITD translation by preventing the binding of polypyrimidine tract-binding protein 1 (PTBP1) from FLT3 messenger RNA and impaired the cytoactivity of inhibitor-resistant FLT3-ITD AML, suggesting that circMYBL2 knockdown was effective against FLT3-ITD AML with quizartinib resistance [125]. Notably, circRNAs can regulate the assembly of membraneless organelles to overcome drug resistance [40, 189]. For example, circRNA-CREIT facilitates the interaction between PKR and the E3 ligase HACE1 to promote proteasomal degradation of PKR, which attenuates the assembly of stress granules (SGs) to activate the RACK1/MTK1 apoptosis signaling pathway and overcome doxorubicin resistance in TNBC [40] (Fig. 6d).

Drug resistance is an urgent problem to be solved in current tumor therapy treatments. Recent studies have shown that circRNAs can regulate drug tolerance pathways by interacting with miRNAs, proteins and translated proteins in tumor cells [33, 130, 189]. Targeting drug resistance-related circRNAs may improve the efficiency of chemotherapeutics in cancers.

## Challenges of circRNAs as therapeutic targets

Although recent studies have suggested that circRNAs are promising therapeutic targets in many diseases, there are still some challenges [67, 99, 130]. Currently, two targeted therapies are commonly used: gene editing systems and RNAi [123, 141–143, 146]. The gene editing method uses the CRISPR‒Cas9 system to specifically delete the *Alu* sequence, which is important for circRNA formation [4, 10, 15, 143]. Such an operation does not affect the mRNA content of the corresponding linear product of the gene but only affects the formation of circRNA, thus regulating the life activities of the cell. However, this method often leads to the occurrence of unpredictable selective shearing events, and DNA editing is an irreversible operation with potential ethical problems. On the contrary, RNAi technology is relatively safe to change cellular RNA levels for it will not cause gene changes [67, 125, 141, 195–197]. It induces circRNA cleavage by delivering small interfering RNA or short hairpin RNA to cells and reduces the content of circRNA. In addition, the CRISPR‒Cas13 system is increasingly being utilized to effectively target circRNA without affecting mRNA and has been shown to have an overall advantage in the efficiency and specificity of circRNA knockdown [124, 126, 141]. However, the efficiency of introducing gRNA and Cas13 enzymes into target cells is not high, and there is a certain off-target effect. For CRISPR‒Cas13 technology to be truly applied to clinical practice, these problems still need to be further solved.

## Therapeutic potential based on circular RNA translation

**Fig. 7:**
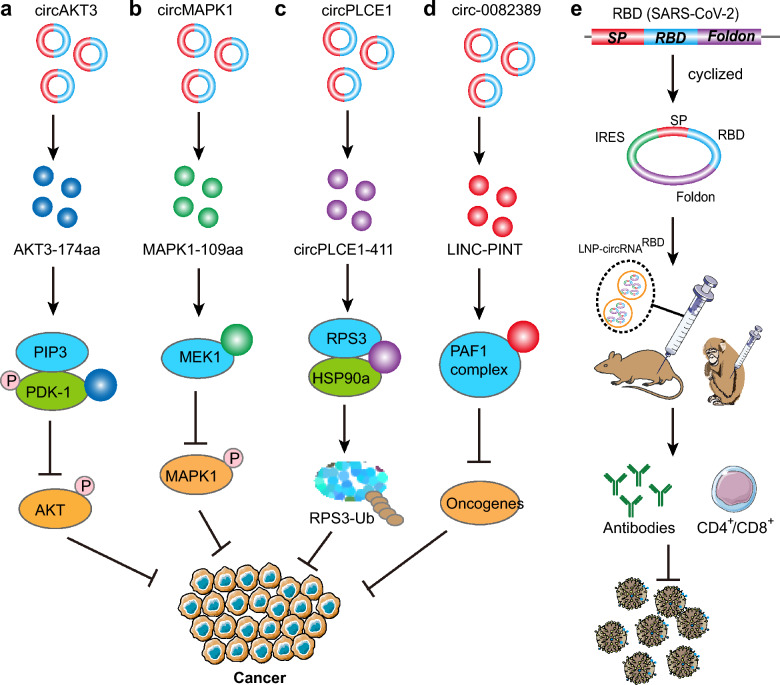
Therapeutic potential based on circRNA translation. **a** circAKT3 encodes a small 174-amino acid peptide, AKT3-174aa, which competitively binds p-PDK1 to inhibit downstream targets of AKT signaling pathway in glioblastoma. **b** MAPK1-109aa, encoded by circMAPK1, can inhibit the phosphorylation of MAPK1 by competitively binding to MEK1, thereby suppressing MAPK pathway and inhibits the proliferation and migration of gastric cancer cells. **c** circPLCE1 encodes circPLCE1-411 that promotes the ubiquitin-dependent degradation of the critical NF-κB regulator RPS3 via directly binding the HSP90α/RPS3 complex to facilitate the dissociation of RPS3 from the complex, thereby reducing NF-κB nuclear translocation in CRC cells. **d** circ-0082389 encodes a small peptide, LINC-PINT, interacts with polymerase associated factor complex (PAF1c) and inhibits the transcriptional elongation of multiple oncogenes in glioblastoma tumorigenesis. **e** A workflow of circRNA-RBD vaccine. SP, signal peptide sequence of human tPA. Foldon, the trimerization domain from bacteriophage T4 fibritin. The circRNARBD-Delta vaccine based on lipid nanoparticle (LNP), which is injected into mice and rhesus macaque, and elicits potent neutralizing antibodies and T cell responses, providing robust protection against SARS-CoV-2

Recent studies have found that some circRNAs can also be directly translated into small peptides and play a role in cells [9, 65, 198]. Interestingly, a number of circRNAs can encode carcinogenic or cancer-inhibiting protein products [199–201] (Fig. 7). For example, circAKT3 has a predicted ORF and encodes a small 174-amino acid peptide, AKT3-174aa, which competitively binds p-PDK1 to inhibit downstream targets of p-PDK1, suppressing glioblastoma tumorigenicity [199] (Fig. 7a). MAPK1-109aa, encoded by circMAPK1, can inhibit the proliferation and migration of gastric cancer cells [200] (Fig. 7b). circPLCE1-411 promotes the ubiquitin-dependent degradation of the critical NF-κB regulator RPS3 by directly binding the HSP90α/RPS3 complex to inhibit the NF-κB signaling pathway in colorectal carcinoma (CRC) [201] (Fig. 7c). In vivo experiments showed that circular LINC-PINT and vSP27 could inhibit the growth of cancer and had no adverse effects on mice [202, 203] (Fig. 7d).

Given that circRNAs have the perfect characteristics of stable conformation, high stability, and special immunogenicity, RNA circle-based technologies were developed [9, 18, 67]. Recently, circRNAs harboring the translational capability of SARS-CoV-2 receptors were used to generate mRNA vaccines, such as the circRNA-RBD-Delta vaccine, which was used to protect against the COVID-19 pandemic [44] (Fig. 7e). However, few studies have investigated circRNAs with mRNA-based therapeutics in cancer treatment. It is a promising strategy to synthesize translational circRNAs with antineoplastic genes in cancer therapy. Similar to small antisense oligonucleotides, efficient introduction of circRNA into target cells is key to clinical implementation. To improve the delivery efficiency of circRNA delivery boxes, vectors can be replaced with lentiviruses or adeno-associated viruses [28, 54, 190, 196]. circRNA expression boxes in target cells may produce a large number of linear products in addition to target circRNA, which may adversely affect cells. We may directly introduce circRNA, which has been synthesized in vitro, into the target cells and deliver it with nonviral nanoparticles [45, 130, 192, 204]. However, in vitro circularized RNAs generally induce extra coding genes or sequences and often activate remarkable immune responses and other unknown side effects. Therefore, future studies may develop specific and effective approaches to improve circular RNA-based therapeutics.

## Conclusions and perspectives

With advances in bioinformatics and biotechnologies, circRNA research has become an increasingly popular and important field [2, 5, 9, 10, 50, 99, 130]. There are many new insights into aspects of circRNA studies, including biogenesis, epigenetic regulation and degradation [4, 5, 9, 10, 67]. Increasing evidence has revealed that circRNAs have dysregulated expression patterns and diverse regulatory mechanisms underlying cellular processes and are always related to the pathogenesis of various diseases, including cancer [20, 130]. However, the study of the regulation, functions and biomedical application of these molecules is still at an early stage, and the complexity of circRNA already appears. For example, diverse biogenesis mechanisms of circRNAs are still emerging. Most annotated circRNAs are produced by back-splicing of pre-mRNA or intron self-splicing of small RNAs [5, 13, 148, 149]. With advances in deep sequencing, especially the development of long-read sequencing, a majority of novel circRNAs are generated by unknown splicing and differential locations on chromatin, such as from incomplete introns or exons with splicing complexity [100, 102, 127]. Some circRNAs were derived from intergenic sequences [50, 205]. The factors regulating these unknown production mechanisms of circRNA should be further delineated. In addition, although many significant advances in identification tools of circRNAs have appeared, it is still difficult to precisely define their length, location, and expression, which are always different from those in experimental validations. This is an important and challenging task in this field, which requires scientists to work together. Advanced parallel technologies will be helpful for circRNA discovery. Some open friendly comprehensive pipelines, such as Fcirc, may offer platforms for users to optimize the discovery tools of circRNAs [64, 89, 92].

The sequence overlaps of circRNAs with their cognate linear RNA sequences usually restrict the determination of circRNA functions [5, 11]. Although recent progress in biotechnologies for knockdown and knockout has been made, uncertain efficiency and off-targeting in si/shRNA or CRISPR/Cas series systems always occur. A recent design based on CRISPR‒Cas13 systems can improve the specificity of targeting BSJ sites [124, 129, 140, 141]. However, the efficiency of expression of Cas13 and sgRNA together is low in cells, especially in cells in suspension, which may restrict their widespread application. Importing some extra sequences and immunogenicity are two difficulties in circRNA overexpression in cells, which affect the application of circRNAs in biomedicine [18, 149]. Novel strategies for circRNA overexpression are urgently needed. In vitro synthesized circRNAs via T4 RNA ligase without extraneous fragments that present minimized immunogenicity may be developed to be a useful method to meet the sufficient quantity of circRNAs in biomedical applications [128].Kindly check and confirm the section headings are correct.Yes, we check and confirm the section headings are correct.

Considering the structural stability advantages, cancer-specific expression, and drug resistance exhibited by circRNAs, they hold significant promise as noninvasive biomarkers for cancer and as targets in cancer treatment [20, 67, 99, 130]. Nonetheless, in clinical practice, the challenge lies in determining the extraction and processing methods for test substances, hindering the quest to establish circRNA as the quickest and most precise biopsy marker for clinical assessments. Additionally, achieving precise in vivo delivery of si/shRNA-based knockdown or tumor suppressor circRNAs in anticancer therapy should be continually optimized. We hope that these issues can be addressed in future research.

The discovery of circRNA translation not only brings exciting new perspectives for translation machines but also brings novel design concepts for the treatment of major diseases based on circRNA translation [32, 62, 206]. The considerable intra- and extracellular stability of circRNA seems to make it a more ideal tool than other ncRNAs in many aspects of biomedical applications [62, 67]. A novel SARS-CoV-2 vaccine based on circRNA-RBD translation was able to produce a higher and longer-lasting antigen and induce a higher proportion of neutralizing antibodies than an mRNA vaccine [44]. However, circRNA-based protein translation strategies are still in the exploratory stage. Many problems remain unresolved. The most important problem is that the translation efficiency of circRNA based on IRES is low. Therefore, the common translational elements of circRNA need to be further optimized. For example, a team found that five elements upstream of the IRES topology, the 5′ PABP spacer, the HBA1 3′ UTR and the HRV-B3 IRES with proximal loop Apt-eIF4G insertion, can considerably improve the translational efficiency of circRNA in vivo [30]. In addition, the search for candidate proteins suitable for circRNA translation strategies should also be continued. A precision medicine approach based on personalized circRNA construction-candidate target-host may be possible in the future. The emergence of circRNA-based protein translation strategies has brought new directions to the field of biomedicine.

## Data Availability

Not applicable.

## Abbreviations

circRNAs: Circular RNAs
ncRNAs: Non-coding RNAs
mRNA: Messenger RNA
lncRNA: Long noncoding RNA
snoRNA: Small nucleolar RNA
miRNA: MicroRNA
ssRNAs: Single-stranded RNAs
tRNAs: Transfer RNAs
pre-mRNA: Precursor mRNA
BSJ: Back-splicing junction site
ICSs: Intronic complementary sequences
RBPs: RNA-binding proteins
RNase R: Ribonuclease R
m6A: N(6)-Methyladenosine
COVID-19: Corona Virus Disease 2019
snRNAs: Small nuclear RNAs
rRNAs: Ribosomal RNAs
RNA-seq: RNA sequencing
UTRs: Untranslated regions
ciRNAs: Intronic circRNAs
EIciRNAs: Exon‒intron circRNAs
ecircRNAs: Exonic circRNAs
circR-loops: CircRNA:DNA hybrids
IRS2: Insulin receptor substrate 2
EGFR: Epidermal growth factor receptor
PABPN1: Nuclear poly (A) binding protein 1
HuR: Human antigen R
dsRNA: Double-stranded RNA
PKR: Double-stranded RNA-activated protein kinase
DRIP-seq: DNA:RNA immunoprecipitation sequencing
R-loop: DNA:RNA hybrids
SARS-CoV-2: Severe Acute Respiratory Syndrome Coronavirus 2
gRNAs: Guide RNAs
ADARs: Adenosine Deaminase
IRES: Internal ribosome entry
elF4: Eukaryotic translation initiation factor 4
elF4G: Eukaryotic translation initiation factor 4G
elF3: Eukaryotic translation initiation factor 3
elF4G38: Eukaryotic translation initiation factor 4G38
eIF4E: Eukaryotic translation initiation factor 4E
Ago2: Argonaute 2
DBR1: Debranching RNA Lariats 1
RNase H1: Ribonuclease H1
SLE: Systemic lupus erythematosus
RNase L: Ribonuclease L
f-circRNAs: Fusion circular RNAs
ceRNA: Competing endogenous RNA
RT: Reverse transcription
PCR: Polymerase Chain Reaction
qPCR: Quantitative PCR
ddPCR: Droplet digital PCR
CCD: Charge-coupled device camera
ISH: In situ hybridization
siRNA: Small interfering RNA
shRNA: Short hairpin RNA
ASO: Antisense oligonucleotides
CRISPR: Clustered Regularly Interspaced Short Palindromic Repeats
GCN1: GCN1 Activator Of EIF2AK4
sgRNAs: Small guide RNAs
td: T4 thymidylate synthase
RIG-I: Retinoic-acid-inducible gene I
SUZ12: SUZ12 polycomb repressive complex 2 subunit
MYBL2: V-Myb avian myeloblastosis viral oncogene homolog-like 2
CLIP-seq: Crosslinking immunoprecipitation-high-throughput-sequencing
RIP: RNA immunoprecipitation
MFNNs: Matrix factorization and neural networks
dRNH1: Human RNase H1
DRIP-seq: DNA:RNA hybrid immunoprecipitation and sequencing
SMARCA5: SWI/SNF Related, Matrix Associated, Actin Dependent Regulator of Chromatin, Subfamily A, Member 5
DSBs: Double-strand DNA breaks
XPO4: Exportin 4
AML: Acute Myelocytic Leukemia
ALL: Acute Lymphocytic Leukemia
CLL: Chronic Lymphocytic Leukemia
CML: Chronic Myeloid Leukemia
MM: Multiple Myeloma
CRC: Colorectal Carcinoma
HCC: Hepatocellular Carcinoma
GC: Gastric Carcinoma
BC: Bladder Cancer
PC: Pancreatic Cancer
OSCC: Oral Squamous Cell Carcinoma
ESCC: Esophageal Squamous Cell Carcinoma
EC: Esophagus Cancer
LC: Lung Cancer
RC: Renal Carcinoma
GM: Glioma Malignancy
OC: Ovarian Cancer
TC: Thyroid Cancer
CC: Cervical Cancer
CEA: Carcinoembryonic antigen
LAA: Large-artery atherosclerosis
TNBC: Triple-negative breast cancer
PDX: Patient-Derived Xenograft
ASOs: Antisense oligonucleotides
TOP1: DNA topoisomerase I
CPT: Camptothecin
FLT3: FMS-like tyrosine kinase 3
ITD: Internal tandem duplication
YBX1: Y-box binding protein 1
PRP19: Pre-mRNA processing factor 19
METTL3: Methyltransferase Like 3
NSCLC: Non-Small Cell Lung Carcinoma
DDP: Cisplatin
GRB7: Growth factor receptor bound protein 7
FAK: Focal Adhesion Kinase
AKT: Protein Kinase B, PKB
ERK: Extracellular regulated protein kinases
HER2: Human epidermal growth factor receptor 2
PTBP1: Polypyrimidine tract-binding protein 1
HACE1: HECT domain and ankyrin-repeat-containing E3 ubiquitin-protein ligase 1
SGs: Stress granules
RACK1: Receptor for Activated C kinase1
MTK1: Mitogen-activated protein kinase kinase kinase 4
RNAi: RNA interference
ORF: Open reading frame
PDK1: 3-Phosphoinositide-dependent protein kinase 1
NF-κB: Nuclear factor kappa-B
RPS3: Ribosomal protein S3
HSP: Heat Shock Proteins
PABP: Poly A binding protein
HBA1: Hemoglobin Subunit Alpha 1
